# Clinical efficacy and safety of bone cement combined with radiofrequency ablation in the treatment of spinal metastases

**DOI:** 10.1186/s12883-020-01998-5

**Published:** 2020-11-18

**Authors:** Nanning Lv, Rui Geng, Feng Ling, Zhangzhe Zhou, Mingming Liu

**Affiliations:** 1 Department of Orthopedic Surgery, The Second People’s Hospital of Lianyungang, 41 Hailian East Street, Lianyungang, 222003 Jiangsu China; 2 School of Public Health, Nanjing Medical University, Nanjing, 211166 Jiangsu China; 3 Department of Orthopedic Surgery, The Taizhou People’s Hospital, Taizhou, 225300 Jiangsu China; 4 Department of Orthopedic Surgery, The First Affiliated Hospital of Soochow University, 188 Shizi Street, Suzhou, 215006 Jiangsu China

**Keywords:** Percutaneous kyphoplasty, Bone cement, Radio frequency ablation, Spinal metastases

## Abstract

**Background:**

To investigate the clinical efficacy and safety of bone cement combined with radiofrequency ablation (RFA) in the treatment of spinal metastases.

**Methods:**

The medical records of patients with spinal metastatic tumor admitted to our hospital from January 2016 to December 2018 were retrospectively analyzed. Based on different surgical methods, the patients were divided into groups A (treated with RFA combined with bone cement) and B (treated with bone cement only). Group A included 35 patients with 47 segments of diseased vertebral bodies. Group B consisted of 52 patients with 78 vertebral segments. Pain, quality of life score, vertebra height, bone cement leakage, postoperative tumor recurrence, and complications were assessed 3 days and 1 and 6 months after surgery.

**Results:**

All the patients had smooth operation without paraplegia, spinal cord injury, and perioperative death. Visual analogue scales (VAS) and Oswestry Disability Index (ODI) scores of the two groups significantly improved 3 days and 1 month after surgery compared with those before surgery (*P* < 0.05), but no significant difference was observed between the two groups (*P* > 0.05). Six months after surgery, the VAS and ODI scores of patients in group A were lower than those in group B, with statistically significant differences (*P* < 0.05). The postoperative vertebral body height of the two groups significantly increased compared with that before surgery, and the difference was statistically significant (*P* < 0.05). Meanwhile, no significant difference was observed between the two groups (*P* > 0.05). Postoperative bone cement permeability in group A was 6.4%, and postoperative tumor recurrence rate was 11.4%. The permeability of bone cement in group B was 20.5%, and the tumor recurrence rate was 30.8%. The bone cement permeability and tumor recurrence rate in group A were lower than those in group B, with statistically significant differences (*P* < 0.05).

**Conclusions:**

Bone cement combined with RFA for the treatment of spinal metastases can achieve good efficacy, desirable analgesic effect, low incidence of complications, small surgical trauma, and high safety. The proposed method has the value of clinical popularization and application.

## Background

The skeletal system is a common metastasis site of malignant tumors, and 70% of patients with advanced malignant tumors develop bone metastasis. About 50% of metastatic bone cancers occur in the spine [1]. Osteolytic destruction most commonly occurs in patients with metastatic spinal cancer. Pathological fracture of the spine is caused by tumor invasion of the vertebral body and its appendages, resulting in severe pain in the lower back, spinal dysfunction, neurological dysfunction, and paraplegia, seriously affecting the quality of life [2]. Traditional surgery or chemoradiotherapy for spinal metastatic cancer causes severe trauma and various complications and has narrow application range and poor efficacy [3].

Percutaneous vertebroplasty (PVP) and percutaneous kyphoplasty (PKP) are commonly used in patients with vertebral metastatic carcinoma to enhance the strength of the vertebral body with bone cement injection, relieve the patients’ pain, and improve the patients’ quality of life, However, defects, such as bone cement leakage and poor tumor control, occur [4, 5]. Radiofrequency ablation (RFA) is a new technique for minimally invasive hyperthermia that effectively prevents tumor cell proliferation. However, RFA can cause cavity formation in the vertebra, reduce the stability of the posterior wall of the vertebra, and increase the fracture risk [6].

Bone cement and RFA have their own advantages in the treatment of metastatic tumors of the spine. This study investigated the clinical efficacy and safety of RFA combined with PVP or PKP in the treatment of patients with spinal metastatic tumor.

## Methods

### Inclusion and exclusion criteria

Inclusion criteria: (1) definite diagnosis of spinal metastatic cancer (pathological or cytological diagnosis); (2) structurally intact posterior margin of the vertebral body without nerve root symptoms; (3) thoracic and lumbar vertebral body lesions, which are mainly lesions of osteolytic destruction or mixed destruction; (4) willingness to undergo the proposed procedure (signed informed consent) and relatively good treatment compliance.

Exclusion criteria: (1) incomplete structure of the posterior margin of the vertebral cortex or infiltration of tumor into the dura, accompanied by nerve root symptoms; (2) osteogenic lesions; (3) terminal patients; (4) severe cardiopulmonary disease or coagulation dysfunction.

### General information

The medical records of patients with spinal metastatic tumor admitted to our hospital from January 2016 to December 2018 were retrospectively analyzed. In accordance with the inclusion and exclusion criteria, 87 patients with 125 vertebral bodies were included. The patients were divided into two groups based on the different surgical methods. Group A: RFA combined with PKP or PVP was used in 35 patients with 47 diseased vertebral bodies. Group B: A total of 52 patients with 78 diseased vertebral bodies underwent PKP or PVP surgery. Table 1 shows the basic information of the two groups. No statistically significant difference was observed between the two groups in terms of age, gender, and disease types (*P* > 0.05), suggesting comparability.

**Table 1:** General and basic information of patients in the two groups

		Group A (*n* = 35)	Group B (*n* = 52)	Test statistics	*P* value
Age(y)		51.4 ± 9.3	52.2 ± 8.5	t = 0.41	0.680
Gender	Male	21	32	χ2 = 0.02	0.885
	Female	14	20		
Surgical levels	Thoracic	26	42	χ2 = 0.03	0.873
	Lumbar	21	36		
Primary lesions	Lung	21	30	–	*P* > 0.05
	Breast cancer	7	13		
	Prostate gland	2	5		
	Kidney	1	1		
	Liver	2	2		
	Lymphatic	1	0		
	Thyroid gland	1	3		

### Surgical technique

Group A (RFA combined with PKP or PVP): The patients were placed on the prone position, the skin diameter of the sterilized operation area was about 40 cm, sterile towel was laid, and local anesthesia was performed at the puncture point with lidocaine. Under the C-arm X-ray machine, the fluoroscopy position was roughly the needle point and needle direction. We can determine the position and angle of the needle from the C-arm X-ray machine. The puncture needle core was removed after the puncture needle reached the vertebral body through the pedicle under imaging monitoring. Electrodes of different specifications were selected depending on the tumor location and size, and the umbrella-shaped electrode needle was inserted into the lesion along the puncture channel. When RFA was performed on the anterior half of the vertebral body, the temperature was set at 90 °C, and the work time was 1.5–2.5 min. When RFA was performed on the posterior part of the vertebral body, the setting temperature was appropriately lowered to 75 °C, the work time was 2.5–3.5 min, the power was 100 ~ 150 W, and the treatment time was 4–6 min. RFA was performed from different angles, and the electrode was removed slowly after the treatment. After RFA, PVP or PKP was performed. PKP was operated to insert the balloon along the original working channel, and the balloon was slowly expanded under imaging monitoring. After the dough stage, the balloon was removed, and the bone cement was slowly pushed into the cavity of the vertebral body under imaging monitoring to prevent the bone cement from leaking outside the vertebral body. In general, 2–5 ml bone cement is suitable for injection. The needle core was inserted and pulled out slowly along with the working passage sleeve to prevent the bone cement from leaving the tail and to cover the sterile dressing. PVP surgery required no balloon implantation, and the remaining procedures were consistent with PKP surgery. After the operation, vital signs are monitored by electrocardiogram, and the patient lay in supine position for 6 h.

Group B (PKP or PVP): The procedure of this group was similar to that of group A (RFA combined with PKP or PVP), but no RFA of tumor tissue was performed. In other words, after the puncture location of the surgical area, the surgeon directly performed PVP or PKP surgery and injected bone cement into the diseased vertebra. After the operation, the operating passage was exited, and the skin puncture was sutured.

Postoperative observation and management: Postoperative puncture point wounds, related nerve functions, and vital signs of patients in the two groups were observed. Postoperative X-ray or computed tomography examination was performed to determine the distribution of bone cement and the presence or absence of bone cement leakage. Symptomatic support treatment was adopted if bone cement leakage occurred. Postoperative patients should stay in bed for 6 h as much as possible and pay attention to analgesic measures. The principle of analgesia is based on the principle of the three steps for cancer pain of World Health Organization. The imaging data of patients were followed up to evaluate the tumor inactivation.

### Assessed parameters

#### Visual analogue scales (VAS)

A score of 0 represents no pain, and 10 denotes severe pain. The patients rated their pain.

#### Oswestry disability index (ODI)

The scale included 10 items: pain, washing and dressing, weightlifting, walking, sitting, standing, sleeping, sexual life (if possible), social activities, and travel. Each part contained six options, representing 0–5 points. The lower the score, the more accessible the life.

#### Recovery of vertebral body

Preoperative and postoperative vertebral anterior margin and central height were measured on X-ray film to observe the orthopedic condition of the diseased vertebra.

The leakage of bone cement was evaluated by X-ray examination.

### Statistical methods

SPSS19.0 statistical software was used for data analysis. Measurement data were expressed as mean ± standard deviation, two-sample independent t test was used for inter-group comparison, and counting data were expressed as rate (%). *P* < 0.05 indicated statistically significant difference.

## Results

The pain of the two groups was alleviated 1 day and 1 month after surgery compared with that before surgery, and the difference was statistically significant (*P* < 0.05). However, no significant difference was observed between the two groups (*P* > 0.05). Six months after surgery, the pain score of the bone cement combined with RFA group was lower than that of the bone cement only group, with a statistically significant difference (*P* < 0.05) (Table 2).

**Table 2:** VAS scores of the two groups before and after surgery

	Group A (*n* = 35)	Group B (*n* = 52)	t value	*P* value
Preoperative	7.52 ± 1.44	7.63 ± 1.52	0.34	0.736
After 3 days	2.79 ± 0.53*	2.88 ± 0.51*	0.79	0.429
After 1 month	2.14 ± 0.40*	2.28 ± 0.43*	1.53	0.130
After 6 months monthmonths	2.23 ± 0.46*	3.15 ± 0.52*	8.47	< 0.001

*: Compared with the preoperative results, the difference was statistically significant (*P* < 0.05)

No significant difference was noted in the preoperative ODI score between the two groups (*P* > 0.05). At 1 day and 1 month after surgery, the ODI score of patients in the two groups was significantly lower than that before surgery, and the difference was statistically significant (*P* < 0.05). However, no significant difference was recorded between the two groups (*P* > 0.05). Six months after the operation, the ODI score of the bone cement combined with the RFA group was lower than that of the bone cement alone group, and the difference was statistically significant (*P* < 0.05) (Table 3).

**Table 3:** ODI scores of patients in the two groups before and after surgery

	Group A (*n* = 35)	Group B (*n* = 52)	t value	*P* value
Preoperative	77.52 ± 8.84	76.65 ± 8.12	0.47	0.638
After 3 days	48.79 ± 6.45*	49.42 ± 6.94*	0.43	0.671
After 1 month	43.23 ± 5.69*	45.08 ± 6.43*	1.38	0.172
After 6 months	46.46 ± 6.46*	52.15 ± 7.52*	3.66	< 0.001

*: Compared with the preoperative results, the difference was statistically significant (*P* < 0.05)

Height of anterior vertebral edge: No significant difference was observed in the preoperative height of anterior vertebral edge between the two groups (*P* > 0.05). The postoperative anterior vertebral height of the two groups was significantly higher than that before surgery, and the differences were statistically significant (*P* < 0.05). However, no significant difference was observed between the two groups (*P* > 0.05) (Table 4).

**Table 4:** Anterior height of vertebral body before and after surgery in the two groups(mm)

	Group A (*n* = 47)	Group B (*n* = 78)	t value	*P* value
Preoperative	18.53 ± 3.84	18.66 ± 3.24	0.20	0.840
Postoperative	24.23 ± 4.25	23.89 ± 4.34	0.43	0.670
t value	6.81	8.53	–	–
*P* value	< 0.001	< 0.001	–	–

Intermediate height of vertebral body: The two groups showed no statistically significant difference in terms of preoperative intermediate height of the vertebral body (*P* > 0.05). The middle height of vertebral body in the two groups increased significantly after surgery compared with that before surgery, and the difference was statistically significant (*P* < 0.05). However, no significant difference was observed between the two groups (*P* > 0.05) (Table 5).

**Table 5:** Intermediate height of vertebral body before and after surgery of patients in the two groups (mm)

	Group A (*n* = 47)	Group B (*n* = 78)	t value	*P* value
Preoperative	24.12 ± 3.88	24.32 ± 3.52	0.30	0.768
Postoperative	28.18 ± 4.25	27.33 ± 4.39	1.06	0.291
t value	4.84	3.67	–	–
*P* value	< 0.001	< 0.001	–	–

Bone cement leakage: In group A, three cases of bone cement leakage in vertebral body, including two cases of paravertebral leakage and one case of disc leakage, occurred after surgery, and the rate of bone cement leakage was 6.4%. In group B, 16 cases of bone cement leakage in vertebral body occurred after surgery, and the rate of bone cement leakage was 20.5%. Among these cases, nine were paraspinal leakage, two were disc leakage, four were pedicle leakage, and one was mixed leakage (including two or more types of leakage). The difference in bone cement exosmosis rate between the two groups was statistically significant (*P* < 0.05). The bone cement exosmosis rate significantly reduced in the bone cement combined with the RFA group. Although a small number of patients had bone cement leakage after surgery, they exhibited no symptoms and required no further treatment (Table 6).

**Table 6:** Bone cement leakage and tumor recurrence after surgery in the two groups

		Group A	Group B	χ2 value	*P* value
Bone cement leak	Yes	3	16	4.54	0.033
	No	44	62		
Tumor recurrence	Yes	4	16	4.42	0.036
	No	31	36		

Postoperative tumor recurrence rate: A total of 4 cases of tumor recurrence occurred in group A, whereas 16 were noted in group B, with recurrence rates of 11.4% of and 30.8%, respectively. The difference in the postoperative tumor recurrence rates between the two groups was statistically significant (*P* < 0.05), whereas the tumor recurrence rate in group A was significantly reduced (Table 6).

The comparison of PKP and PVP in groups A and B showed that the recovery of vertebral height in patients with PKP was better than that in patients with PVP (*P* < 0.05). However, no significant difference was observed between cement leakage and recurrence (*P* > 0.05) (Tables 7 and 8).

**Table 7:** The patients were treated with RFA combined with PKP or PVP

	PVP (*n* = 27)	PKP (*n* = 20)	*P* value
AVH			
Preoperative	18.49 ± 3.86	18.60 ± 3.75	0.923
Postoperative	23.54 ± 4.22	25.16 ± 4.12	0.195
IVH			
Preoperative	24.02 ± 3.73	24.26 ± 3.68	0.827
Postoperative	25.92 ± 4.02	29.23 ± 4.29	0.009
Bone cement leak	4(14.8%)	2(10.0%)	0.962
Tumor recurrence	2(7.4%)	2(10.0%)	0.831

*AHV* Anterior height of vertebral body, *IHV* Intermediate height of vertebral body

**Table 8:** The patients were treated with PKP or PVP

	PVP (*n* = 40)	PKP (*n* = 38)	*P* value
AVH			
Preoperative	18.74 ± 3.63	18.38 ± 3.25	0.646
Postoperative	22.12 ± 4.31	25.75 ± 4.24	0.000
IVH			
Preoperative	24.35 ± 3.57	24.28 ± 3.38	0.930
Postoperative	25.98 ± 4.12	28.75 ± 4.25	0.005
Bone cement leak	15(37.5%)	7(18.4%)	0.105
Tumor recurrence	7(17.5%)	9(23.7%)	0.692

## Discussion

Metastatic carcinoma of the spine is one of the most common clinical spinal diseases; it is mainly caused by extra-osseous malignant tumors that spread to the spine through hematogenous spread or lymphatic metastasis [7, 8] and characterized by the involvement of multiple vertebral bodies. Metastatic lesions often cause severe pain in patients due to the destruction of the vertebral body by tumor cells, resulting in the fracture or collapse of the vertebral body and inflammation, and by the stimulation of peripheral nerve endings by tumor cells. The key to treating metastatic tumors of the spine is pain relief and prevention of pathologic fractures and other complications that may result from the disease. Currently, PVP and PKP are widely used in the palliative treatment of spinal tumors. Compared with open surgery, these procedures have the advantages of less trauma, short operation time, and less bleeding [9, 10]. Bone cement injected into the vertebra provides support and reduces pain. At the same time, the heat generated by the polymerization of bone cement and the chemical toxicity of bone cement can destroy the nerve endings of tumor tissues and pathological sites. In the past, PVP/PKP was mainly used for the treatment of osteolytic tumors. The application of PVP/PKP in the treatment of osteogenic tumor has increased gradually in recent years. Chen et al. [11] believe that PVP is safe and effective in the treatment of osteoblastic tumors. Wang et al. [12] divided 45 patients with spinal metastatic tumor into the osteoclast and osteogenic groups and performed PKP. The VAS and ODI scores significantly improved in the two groups after surgery, and no statistically significant difference was observed in the postoperative changes in vertebral body height and local kyphosis angle between the two groups. Finally, the said researchers assumed that PKP can effectively relieve pain and improve the dysfunction caused by osteogenic or osteoclastic metastatic tumors of the spine. Bone cement leakage is common in PVP/PKP, with the incidence reaching between 4.8 and 39%; the value may be higher in patients with spinal metastatic cancer [13]. Most cement leakages cause inevident clinical symptoms or mild pain and discomfort, and most patients need not to be treated conservatively with drugs. However, if massive leakage of bone cement into the intervertebral foramina or spinal canal can cause severe pain and paralysis, emergency surgery is required. In recent years, more attention has been paid to the distant leakage of bone cement. Bone cement can also leak into a certain distance through the venous system, resulting in embolization of the heart, lungs, and other organs. Several patients suffer from serious complications and need surgical intervention [14, 15]. At present, polymethyl methacrylate (PMMA) is the most clinically used bone cement. PMMA is not a special bone cement designed for spinal tumors, and it exhibits a poor long-term control effect on local tumors.

RFA is a targeted therapy that is safe and effective in the treatment of liver and lung tumors [16]. In 1992, Rosenthal et al. first proposed the use of RFA to treat osseous lesions; since then, the method had been increasingly used in the management of bony metastases [17]. RFA is an emerging minimally invasive therapy for tumors, aimed at relieving pain and controlling local tumor progression. RFA mainly involves placing the radiofrequency needle into the tumor bone metastasis site through image guidance, and the high-frequency AC current is transmitted to the surrounding tissues through the needle tip, causing tissue fever and cell necrosis. Ablation is controlled based on a feedback system dependent on tissue impedance, which is often high in bone [18]. With RFA procedures, the goal is to induce coagulative necrosis with heat, ideally around 70 °C for bone lesions [19]. Its advantage is rapid cell death and the controllability of ablation range and temperature, However, the ablation area cannot be observed in real time. The use of RFA to assist PVP is safe and reliable. Bone cement was injected into the cavity that formed after RFA, and the leakage of bone cement in the venous and posterior wall of the vertebral body was significantly reduced, which may be related to the formation of thrombus during the ablation process [20]. The mechanism by which RFA reduces pain in bone metastases is unclear. The possible reasons include the following [21]: (1) physical destruction of adjacent sensory nerve fibers into the periosteum and bone cortex, preventing pain transmission; (2) decreased tumor volume to reduce the pressure in sensory nerve fiber stimulation; (3) destruction of tumor cells that produce the nerve stimulating factor; (4) inhibition of osteoclast activities that cause pain.

Given the irregular shape of the tumor in vertebral body, evenly filling the injected bone cement into the vertebral body is difficult. The weak thermal effect of bone cement results in the difficulty in complete elimination of tumor cells, whereas complicated tumor blood vessels are prone to bone cement extravasation. The medium- and long-term tumor recurrence rate and bone cement exosmosis rate of PKP and PVP were high, which indicate the unsatisfactory application effect of PKP. RFA was performed before PVP and PKP, and this step can be more effective in eliminating tumor tissue and clotting and embolizing tumor blood vessels. When bone cement was injected again, the problems of PKP and PVP mentioned above can be avoided to a certain extent. The application of bone cement combined with RFA in the treatment of spinal metastatic cancer can theoretically achieve the effect of combined strength and complementary advantages of the used methods [16, 22]: (1) physical destruction by RFA directly kills cancer cells, can carbonize soft tissue tumors in vertebral bodies, and reduce tumor volume, thus providing a cavity for filling bone cement [23]. RFA can solidify the vascular tissue around the tumor and form microthrombosis in the tumor blood vessel, thus reducing the venous leakage of bone cement during PKP. RFA reduces the pathologic fractures induced by tumor progression and indirectly improves the role of PKP in preventing pathologic fractures [24]. PKP can strengthen the diseased vertebral body after PRF treatment and prevent the pathological fracture of vertebral body induced by RFA. For vertebral bodies with pathologic fractures, PKP can reset the diseased vertebra and avoid further nerve root pain and paraplegia [25]. In this study, the VAS and ODI scores of the two groups were significantly improved at 3 days and 1 month after surgery, but no significant difference was observed between the two groups. This finding suggests that bone cement treatment alone and bone cement combined with RFA can relieve the patients’ recent pain and improve their quality of life without significant difference. The height recovery of the diseased vertebral body of the two groups of patients improved after surgery compared with before surgery, whereas no significant difference was noted when comparing the two treatment methods. This condition suggests that the two treatments exhibited no significant difference in terms of the correction of the diseased vertebral body. After 6 months of follow-up, the bone cement exosmosis rate and tumor recurrence rate in the bone cement combined with RFA group were lower than those in the single bone cement group. The VAS and ODI scores in the bone cement group at 6 months after operation showed aggravated postoperative pain and decreased quality of life. As a surgical palliative treatment, the effect of bone cement combined with RFA group is significant. The present study suggests that bone cement combined with RFA is superior to bone cement in improving the prognosis and medium-term pain of patients with spinal metastatic cancer.

PKP and PVP have good effects on relieving pain and restoring vertebral height [26]. By comparison, regardless of the group, PKP patients had better vertebral height recovery than PVP patients. However, cement leakage and tumor recurrence showed no difference, which may be related to our small sample size and needs further study.

### Limitations

The present study has several limitations that should be considered. First, our study was a retrospective research with a small sample size and short-term follow-up. Second, the nature of the patients’ primary cancer foci and tumor location and size caused difficulty in finding a completely homogeneous control group. The treatment of spinal metastatic carcinoma with percutaneous RFA combined with PKP needs to be validated and summarized in basic research and long-term clinical follow-up.

## Conclusions

In conclusion, bone cement combined with RFA in the treatment of spinal metastatic tumor can effectively relieve patients’ pain, improve their ability for daily activities, enhance the spinal stability, and improve the safety of bone cement treatment and patient prognosis. The proposed strategy can also be widely used in the clinical treatment of patients with spinal metastatic tumor.

## Data Availability

The datasets used and/or analysed during the current study available from the corresponding author on reasonable request.

## Abbreviations

RFA: Radiofrequency ablation
PVP: Percutaneous vertebroplasty
PKP: Percutaneous kyphoplasty
VAS: Visual analogue scales
ODI: Oswestry Disability Index
